# Recanalization using direct stenting before bowel resection for acute-on-chronic superior mesenteric artery occlusion: A case report

**DOI:** 10.1016/j.ijscr.2020.02.040

**Published:** 2020-02-21

**Authors:** Toshinao Suzuki, Satoru Murata, Taichiro Tsunoyama, Maki Kitamura

**Affiliations:** a Interventional Radiology Center, Teikyo University Chiba Medical Center, 3426-3 Anesaki, Ichihara, Chiba, 299-0111, Japan; b Emergency and Intensive Care Center, Teikyo University Chiba Medical Center, 3426-3 Anesaki, Ichihara, Chiba, 299-0111, Japan

**Keywords:** ACMI, acute-on-chronic mesenteric ischemia, AMI, acute mesenteric ischemia, CMI, chronic mesenteric ischemia, SMA, superior mesenteric artery, CT, computed tomography, Mesenteric ischaemia, Superior mesenteric artery occlusion, Acute-on-chronic, Revascularisation, Endovascular therapy

## Abstract

**Introduction:**

Acute-on-chronic mesenteric ischemia (ACMI) refers to acute mesenteric ischemia (AMI) developing in a patient displaying typical symptoms of chronic mesenteric ischemia (CMI). Delayed treatment can cause short bowel syndrome and increased mortality. Intervention involves intestinal revascularization and resection of the necrotic intestine. However, the revascularization procedure must consider the chronic nature of the occlusion.

**Presentation of case:**

A 79-year-old man presented with periumbilical pain for 6 h. AMI was diagnosed, together with chronic superior mesenteric artery occlusion and suspected intestinal necrosis. The symptomatic CMI might have insufficient blood flow to intestines. Endovascular recanalization of the superior mesenteric artery using direct stenting was performed before laparotomy to improve blood flow to the intestines. Subsequent laparotomy revealed approximately 60 cm of ischemic small bowel extending from the jejunum (300 cm anal to the ligament of Treitz) to the ileum (30 cm oral to the terminal ileum). The necrotic bowel was resected without anastomosis. At the second-look operation, further resection was not required.

**Discussion:**

Making a differential diagnosis between acute and acute-on-chronic occlusions is essential for determining the necessity of recanalization and the method of restoring the intestinal blood flow. Here, the patient with symptomatic CMI might have had insufficient blood flow to the intestines despite establishing collateral supply. We determined that recanalization was needed. Direct stenting without predilation could save time to recanalization and result in less risk of distal embolization.

**Conclusion:**

This case suggests prompt recanalization using direct stenting can minimize subsequent bowel resection in patients with ACMI.

## Highlights

• Early notice of acute-on-chronic mesenteric ischemia leads to improved survival.
• Endovascular recanalization and surgery may be effective for this type of ischemia.
• Direct stenting could save time of recanalization.
• As recanalization time is saved, it leads to less distal embolization risk.

## 1 Introduction

Acute-on-chronic mesenteric ischemia (ACMI) is defined as acute mesenteric ischemia (AMI) occurring in a patient who has previously displayed typical symptoms of chronic mesenteric ischemia (CMI) [1]. Both ACMI and AMI are critical conditions with a high mortality rate that require immediate diagnosis and treatment [[1], [2], [3]]. Delayed diagnosis and treatment of intestinal necrosis associated with mesenteric ischemia can lead to short bowel syndrome and increased mortality. For patients with AMI, either open or endovascular revascularization should be considered prior to bowel surgery [1]. However, revascularization can be problematic in patients with ACMI because chronic occlusion is more difficult to treat than fresh occlusion is. In addition, the most effective management of ACMI for reducing mortality and improving gastrointestinal outcomes is unclear. We report our experience with a patient who had acute-on-chronic occlusion of the superior mesenteric artery (SMA) and suspected bowel necrosis. The treatment included prompt endovascular recanalization prior to bowel resection and postoperative open abdomen management. The following case report has been written as per the SCARE guidelines [4].

## 2 Case presentation

A 79-year-old man with a history of radical prostatectomy for prostate cancer and hypertension presented to the emergency department with periumbilical pain persisting for approximately 6 h. He had also noted recurrent postprandial dull epigastric pain.

On admission, he was afebrile, his blood pressure was 206/179 mmHg, his heart rate was 50 beats/min (regular), and his oxygen saturation was 99 % of room air.

**Fig. 1:**
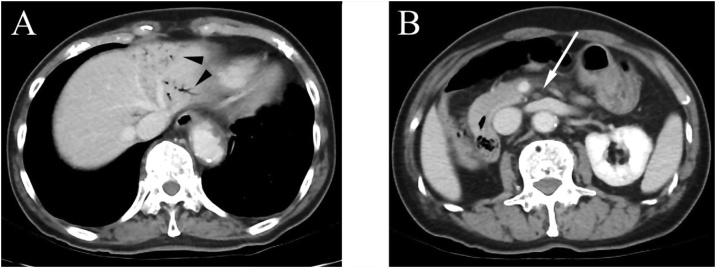
Initial enhanced computed tomography (CT) findings. **A** Black arrowhead: gas in hepatic portal veins. **B** White arrow: complete occlusion of the superior mesenteric artery.

**Fig. 2:**
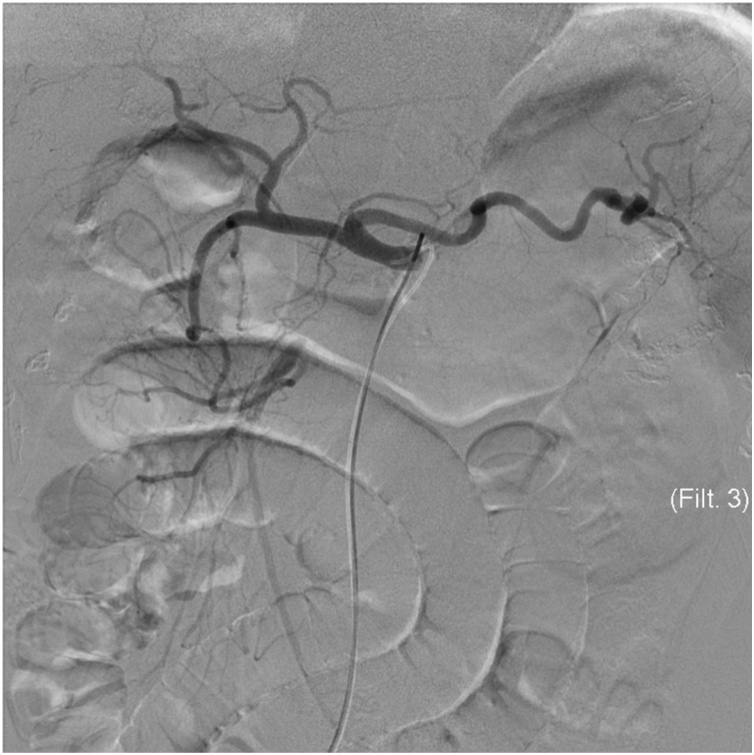
Angiography from the celiac artery shows the collateral pathway that supplies the superior mesenteric artery.

**Fig. 3:**
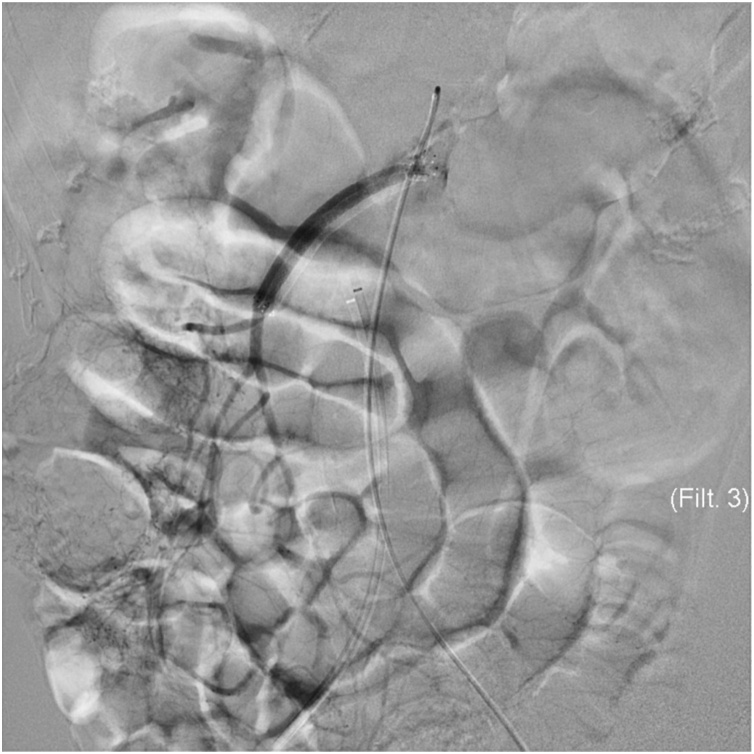
Angiography confirms successful recanalization of the SMA with a 6 mm × 80 mm LifeStent Solo.

Examination revealed that the abdomen was tender to palpation, with rigidity, guarding, and rebound tenderness. Laboratory tests revealed that D-dimer was 2.6 μg/mL, the white blood cell count was 11.2 × 10^9^/L, and C-reactive protein was <0.3 mg/dL (undetectable). His acid-base balance was preserved, but his venous lactate level was 3.7 mmol/L. The chest X-ray result was normal, whereas plain abdominal X-ray result revealed small bowel distension. The electrocardiogram showed sinus rhythm without ST elevation or Q waves. Contrast-enhanced computed tomography (CT) showed presence of gas in hepatic portal veins, and the superior mesenteric vein was larger than the SMA (Fig. 1A). CT also identified a defect at the origin of SMA, and enhancement was observed distal to the main trunk of the artery (Fig. 1B). SMA occlusion was revealed by retrospective assessment of CT scans performed 17 months earlier at the hospital. The CT result was not in the patient’s record, so the details were unknown at presentation. AMI with chronic SMA occlusion was diagnosed. CMI might usually have collateral supply, but symptomatic cases can have minimal blood flow. To secure the intestinal blood flow, endovascular treatment was performed before open surgery. Celiac artery angiography showed collateral vessels from the celiac artery to the distal SMA (Fig. 2). A 0.035-inch guidewire (Terumo, Tokyo, Japan) was advanced into the occluded lesion in the SMA. To place a guiding sheath (Destination®, Terumo, Tokyo, Japan), the guidewire was exchanged for a 180-cm Amplatz Super Stiff Guidewire (Boston Scientific Corporation, Boston, MA, USA) using a 4-Fr Cobra catheter (Terumo, Tokyo, Japan). After this, a self-expandable 6 mm × 80 mm LifeStent Solo (Bard Peripheral Vascular, Tempe, AZ, USA) was deployed without predilation (Fig. 3). The time from puncture to recanalization was 59 min.

Subsequent emergency laparotomy revealed about 60 cm of the ischemic small bowel extending from the jejunum (300 cm anal to the ligament of Treitz) to the ileum (30 cm oral to the terminal ileum). The necrotic bowel was resected without anastomosis. Open abdomen management was selected to monitor bowel viability prior to definitive anastomosis and closure. Postoperatively, the patient was admitted to the intensive care unit. When a second-look operation was performed two days later, additional resection was not required and small bowel anastomosis was performed. The patient was discharged on dual antiplatelet therapy (aspirin 100 mg qd, clopidogrel 75 mg qd) to prevent in-stent restenosis.

## 3 Discussion

Mesenteric ischemia can be divided into three categories according to its clinical presentation: acute, chronic, and acute-on-chronic ischemia. AMI is defined as a sudden interruption of intestinal blood flow with development of symptoms that may vary in onset time from minutes to hours. The chief symptom is severe abdominal pain. If untreated, AMI can progress to intestinal necrosis and peritonitis [2]. CMI is generally characterized by postprandial pain, weight loss due to fear of eating, or unexplained diarrhea. These ischemic symptoms are caused by insufficient blood supply to the gastrointestinal tract and have a duration of at least 3 months. ACMI is defined as AMI occurring in patients who previously had typical symptoms of CMI [1]. Besides the clinical presentation, the presence or absence of advanced intestinal ischemia (free air, extensive pneumatosis) is important to consider when selecting the treatment method.

It was reported that endovascular intervention may be as effective as standard surgery in patients who have AMI, but not advanced intestinal ischemia [5]. However, patients with AMI and intestinal ischemia should undergo immediate surgical exploration. Management of ACMI is generally the same as that of AMI; however, patients with CMI often have a well-stablished collateral blood supply via the pancreatico-duodenal arcade. The fact that the patient in this case had recurrent, postprandial, dull, epigastric pain indicates insufficient blood flow to the intestines despite the blood supply from collateral vessels formed due to CMI. The need for intestinal revascularization to re-establish the blood flow and resection of the necrotic bowel should be assessed.

In patients with acute thrombotic occlusion of the SMA, it has been reported that endovascular treatment improves mortality and bowel resection rates more than open revascularization does [1]. Nearly 40 % of patients undergoing a second-look operation ultimately needs further bowel resection [3].

However, extensive bowel resection leads to short bowel syndrome that may require long-term parenteral nutrition, and also increases morbidity.

The patient in this case had acute peritonitis; this also included hepatic portal veins gas with image findings; moreover, this appeared to be a condition of intestinal necrosis. The patient had a symptomatic CMI with SMA occlusion. These are potential symptoms and etiology of bowel ischemia and necrosis. The reporting of surgically resected specimen of the ileum showed ischemic change without embolic, mild arteriosclerosis in the mesenteric artery; additionally, there was no tumor mass present. Furthermore, there was no evidence of trauma or infection from the patient’s medical history and clinical course.

Making a differential diagnosis between acute and acute-on-chronic occlusion is essential for determining the necessity of recanalization and the method of restoring the intestinal blood flow. When performing revascularization, ACMI should be managed as chronic occlusion. Stenting or surgical bypass should be used for patients with ACMI or CMI, with the choice varying among institutions [6]. Endovascular intervention can restore perfusion more rapidly than open repair can and may prevent the progression of mesenteric ischemia to bowel necrosis [3]. However, there is a risk that distal embolization due to endovascular treatment may exacerbate intestinal ischemia.

Direct stenting without predilation rather than thrombolysis or thrombectomy can save time of recanalization and result in less risk of distal embolization. Whatever method is selected, revascularization and bowel resection must be performed as emergency procedures.

## 4 Conclusion

The present case provides evidence that prompt endovascular recanalization by direct stenting prior to laparotomy may minimize the extent of bowel resection. Early recognition of acute-on-chronic mesenteric ischemia and its rapid treatment can improve survival and avoid short bowel syndrome.

## Declaration of Competing Interest

None.

## Sources of funding

None.

## Ethical approval

The case report was exempted from ethical approval from the institution.

## Consent

Consent for publication was obtained from the patient. Our manuscript include a specified statement at the end of the manuscript.

## Author contribution

TS – Authored submission with SM. Assisted with embolization procedure.

TT – Patient under care of this physician.

MK – Patient under care of this physician.

SM – Performed embolization procedure.

All authors read and approved the final manuscript.

## Registration of research studies

Not applicable.

## Guarantor

Toshinao Suzuki.

## Provenance and peer review

Not commissioned, externally peer-reviewed.

## References

[1] Björck M., Koelemay M., Acosta S., Bastos Goncalves F., Kölbel T. (2017). Editor’s choice – management of the diseases of mesenteric arteries and veins: clinical practice guidelines of the European Society of Vascular Surgery (ESVS). Eur. J. Vasc. Endovasc. Surg..

[2] Bala M., Kashuk J., Moore E.E., Kluger Y., Biffl W., Gomes C.A. (2017). Acute mesenteric ischemia: guidelines of the World Society of Emergency Surgery. World J. Emerg. Surg..

[3] Clair D.G., Beach J.M. (2016). Mesenteric ischemia. N. Engl. J. Med..

[4] Agha R.A., Fowler A.J., Saetta A., Barai I., Rajmohan S., Orgill D.P., for the SCARE Group (2018). The SCARE statement: consensus-based surgical case report guidelines. Int. J. Surg..

[5] Beaulieu R.J., Arnaoutakis K.D., Abularrage C.J., Efron D.T., Schneider E., Black III J.H. (2014). Comparison of open and endovascular treatment of acute mesenteric ischemia. J. Vasc. Surg..

[6] Kasirajan K., O’Hara P.J., Gray B.H., Hertzer N.R., Clair D.G., Greenberg R.K. (2001). Chronic mesenteric ischemia: open surgery versus percutaneous angioplasty and stenting. J. Vasc. Surg..

